# Supplementary material for: Brain activity in Cluster N and the hippocampus in non-migratory zebra finches completing a spatial orientation task using magnetic compass information
Source: PLoS One. 2026 Apr 30;21(4):e0348066. doi: 10.1371/journal.pone.0348066 (PMC13132197; doi:10.1371/journal.pone.0348066)
Supplement: S1 Table — Included is information on light conditions, the birds’ activity, presence of migratory restlessness and magnetic field conditions during exposure before sacrifice, where available. ZENK/c-Fos expression in cluster N is categorized into 0 (no activation), + (little activation), ++ (moderate activation), and +++ (high activation). (DOCX) [file pone.0348066.s001.docx]

Supplementary Table 1

| Supplementary Table 1. |
| --- |
| Summary of published studies examining immediate early gene expression in Cluster N in songbirds. Included is information on light conditions, the birds activity, presence of migratory restlessness and magnetic field conditions during exposure before sacrifice, where available. ZENK/c-Fos expression in cluster N is categorized into 0 (no activation), + (little activation), ++ (moderate activation), and +++ (high activation). |

| Species | Light | Activity | Migratory restlessness | Magnetic field (MF) | ZENK/c-Fos in Cluster N | Sample size | Comments on behaviour | Comments on circumstances | Comments on setup | Reference |
| --- | --- | --- | --- | --- | --- | --- | --- | --- | --- | --- |
| White-throated sparrow | bright | yes | no | natural | + | 11 | daytime activity | migration season |  | Brodbeck et al. 2023 |
| White-throated sparrow | dim | no | no | natural | + | 11 | sitting still, occ. blinking/moving head | migration season |  | Brodbeck et al. 2023 |
| White-throated sparrow | dim | yes | yes | natural | +++ | 13 |  | migration season |  | Brodbeck et al. 2023 |
| Northern wheatear | dim | no | no | natural | +++ | 2 | sitting still, but awake |  | ~2 mW/m^2^ | Elbers et al. 2017 |
| Northern wheatear | bright | no | no | natural | 0 | 2 | sitting still, but awake |  | ~200 mW/m^2^ | Elbers et al. 2017 |
| Garden warbler | bright | yes | no | natural | + | 5 | daytime activity | migration season |  | Feenders et al. 2008 |
| Garden warbler | dim | no | no | natural | +++ | 11 | sitting still at night | migration season | ~1 mW/m^2^ (moonlight) | Feenders et al. 2008 |
| Garden warbler | bright | no | no | natural | + | 5 | sitting still during day | migration season |  | Feenders et al. 2008 |
| Garden warbler | dim | yes | yes | natural | +++ | 15 |  | migration season | ~1 mW/m^2^ (moonlight) | Feenders et al. 2008 |
| Empidonax flycatchers | bright | * | no | changing | 0 | 5 |  | migration season | 310-632 mW/m^2^; MF changing in all dimensions every 30 s | Gulson-Castillo 2024 |
| Red-eyed vireo | bright | * | no | changing | 0 | 4 |  | migration season | 310-632 mW/m^2^; MF changing in all dimensions every 30 s | Gulson-Castillo 2024 |
| Swainson's thrush | bright | * | no | changing | 0 | 3 |  | migration season | 310-632 mW/m^2^; MF changing in all dimensions every 30 s | Gulson-Castillo 2024 |
| Empidonax flycatchers | dim | * | * | zero | ++ | 5 |  | migration season | 0.17-2.44 mW/m^2^; zero magnetic field | Gulson-Castillo 2024 |
| Empidonax flycatchers | dim | * | * | changing | ++ | 5 |  | migration season | 0.17-2.44 mW/m^2^; MF changing in all dimensions every 30 s | Gulson-Castillo 2024 |
| Red-eyed vireo | dim | * | * | zero | ++ | 3 |  | migration season | 0.17-2.44 mW/m^2^; zero magnetic field | Gulson-Castillo 2024 |
| Red-eyed vireo | dim | * | * | changing | +++ | 2 |  | migration season | 0.17-2.44 mW/m^2^; MF changing in all dimensions every 30 s | Gulson-Castillo 2024 |
| Swainson's thrush | dim | * | * | zero | ++ | 5 |  | migration season | 0.17-2.44 mW/m^2^; zero magnetic field | Gulson-Castillo 2024 |
| Swainson's thrush | dim | * | * | changing | ++ | 5 |  | migration season | 0.17-2.44 mW/m^2^; MF changing in all dimensions every 30 s | Gulson-Castillo 2024 |
| Garden warbler | dark | no | no | natural | 0 | 1 | both eyes covered | migration season | ~1x10-5 mW/m^2^ | Hein et al. 2010 |
| Garden warbler | dim | no | no | natural | +++ | 1 | eyes open | migration season | ~2 mW/m^2^ | Hein et al. 2010 |
| Zebra finch | bright | no | no | changing | +++ | 7 |  |  | horiz. MF changing @ 120°/s | Keary & Bischof 2012 |
| Zebra finch | bright | no | no | natural | ++ | 7 |  |  |  | Keary & Bischof 2012 |
| Garden warbler | dim | yes | yes | natural | +++ | 5 |  | migration season | ~1 mW/m^2^ | Liedvogel et al. 2007 |
| Garden warbler | dim | yes | yes | changing | +++ | 13 |  | migration season | ~1 mW/m^2^; MF changing 120 deg/5 min | Liedvogel et al. 2007 |
| Garden warbler | dim | yes | yes | zero | +++ | 10 |  | migration season | ~1 mW/m^2^; zero magnetic field | Liedvogel et al. 2007 |
| European robin | dark | no | no | changing | + | 5 | sitting still, both eyes covered | migration season |  | Liedvogel et al. 2007 |
| European robin | dim | no | no | changing | +++ | 6 | sitting still, eyes open | migration season |  | Liedvogel et al. 2007 |
| Garden warbler | dim red | no | no | natural | +++ | 4 | sitting still, eyes open | winter | ~1 mW/m^2^; red light | Liedvogel et al. 2007 |
| European robin | dim | no | no | changing | ++ | 8 | sitting still, left eye covered | migration season |  | Liedvogel et al. 2007 |
| European robin | dim | no | no | changing | +++ | 7 | sitting still, right eye covered | migration season |  | Liedvogel et al. 2007 |
| Sardinian warbler | dim | no | no | changing | +++ | 3 | daytime activity | autumn | ~1 mW/m^2^; MF changing 120°/5 min | Liedvogel et al. 2007 |
| Sardinian warbler | bright | yes | no | changing | 0 | 2 | daytime activity | autumn | MF changing 120°/5 min | Liedvogel et al. 2007 |
| Zebra finch | bright | yes | no | natural | 0 | 5 | daytime activity |  | 275 lux (bright indoor light) | Mouritsen et al. 2005 |
| Canary | bright | no | no | natural | 0 | 5 | sitting still, eyes open |  | 275 lux (bright indoor light) | Mouritsen et al. 2005 |
| European robin | dim | no | no | natural | +++ | 4 | sitting still, eyes open | migration season | ~1 mW/m^2^ (moonlight) | Mouritsen et al. 2005 |
| Garden warbler | dim | no | no | natural | +++ | 12 | sitting still, eyes open | migration season | ~1 mW/m^2^ (moonlight) | Mouritsen et al. 2005 |
| Garden warbler | dim | no | no | natural | +++ | 3 | sitting still, eyes open | summer | ~1 mW/m^2^ (moonlight) | Mouritsen et al. 2005 |
| Zebra finch | dim | no | no | natural | 0 | 5 | sitting still, eyes open |  | ~1 mW/m^2^ (moonlight) | Mouritsen et al. 2005 |
| Canary | dim | no | no | natural | 0 | 2 | sitting still, eyes open |  | ~1 mW/m^2^ (moonlight) | Mouritsen et al. 2005 |
| European robin | dark | no | no | natural | + | 5 | sitting still, both eyes covered | migration season |  | Mouritsen et al. 2005, Liedvogel et al. 2007 |
| European robin | dim | no | no | natural | +++ | 6 | sitting still, eyes open | migration season |  | Mouritsen et al. 2005, Liedvogel et al. 2007 |
| Garden warbler | bright | no | no | natural | 0 | 5 | sitting still, eyes open | migration season | 275 lux (bright indoor light) | Mouritsen et al. 2005, Liedvogel et al. 2007 |
| Garden warbler | bright | no | no | natural | 0 | 10 | sitting still, eyes open | migration season |  | Mouritsen et al. 2005, Liedvogel et al. 2007 |
| Black-headed bunting | bright | yes | no | natural | 0 | 6 | daytime activity | migration season | 150 lux | Rastogi et al. 2011 |
| Black-headed bunting | bright | yes | no | natural | +++ | 7 | daytime activity | pre-migratory season | 150 lux | Rastogi et al. 2011 |
| Black-headed bunting | dim | no | no | natural | +++ | 4 | no activity | pre-migratory season | <1 lux | Rastogi et al. 2011 |
| Black-headed bunting | dim | yes | yes | natural | +++ | 6 |  | migration season | <1 lux | Rastogi et al. 2011 |
| Swainson's thrush | bright | yes | no | natural | 0 | 2 | daytime activity | migration season |  | Rudolf et al. 2024 |
| Swainson's thrush | dim | yes | yes | natural | +++ | 4 |  | migration season |  | Rudolf et al. 2024 |
| Zebra finch | dim | yes | no | natural | +++ | 9 |  |  | 27 mW/m^2^ | this study |
| Zebra finch | dim | yes | no | changing | +++ | 7 |  |  | 27 mW/m^2^; horiz. MF changing 120°/s | this study |
| Zebra finch | dim | yes | no | vertical | +++ | 7 |  |  | 27 mW/m^2^; vertical magnetic field | this study |
| Meadow pipit | dim | no | no | natural | +++ | 4 | sitting still, but awake | winter | 4 mW/m^2^ | Zapka et al. 2010 |
| Meadow pipit | bright | no | no | natural | + | 6 | sitting still, but awake | winter | 450 mW/m^2^ | Zapka et al. 2010 |

* large variation in activity between individuals (E. Gulson-Castillo, pers. comm.)

Black-headed bunting (Emberiza melanocephala)

Canary (Serinus canaria)

Empidonax flycatchers (E. flaviventris, E. minimus, E. traillii, E. alnorum)

European robin (Erithacus rubecula)

Garden warbler (Sylvia borin)

Meadow pipit (Antus pretensis)

Northern wheatear (Oenanthe oenanthe)

Red-eyes vireo (Vireo olivaceus)

Sardinian warbler (Sylvia melanocephala)

Swainson's thrush (Catharus ustulatus)

White-throated sparrow (Zonotrichia albicollis)

Zebra finch (Taeniopygio guttata)
