# Supplementary material for: Brain activity in Cluster N and the hippocampus in non-migratory zebra finches completing a spatial orientation task using magnetic compass information
Source: PLoS One. 2026 Apr 30;21(4):e0348066. doi: 10.1371/journal.pone.0348066 (PMC13132197; doi:10.1371/journal.pone.0348066)
Supplement: S1 File — Movement analyses comparing Zenk expression in each Cluster N subregion, and birds’ movement. Analyses of each subregion is also accompanied by a scatter plot with Zenk expression on the y-axis (Zenk immunoreactivity per field of view), and birds’ movement on the x-axis (the total activity of the bird in the 30 minute period, measured in pixels). (DOCX) [file pone.0348066.s002.docx]

Supplemental Analyses

ZEFI Activity analysis

# ClusterN Anterior Ventral Avg

gscatter(data.TotalActivity, data.ClusterNAnteriorVentralAvg, data.Group)


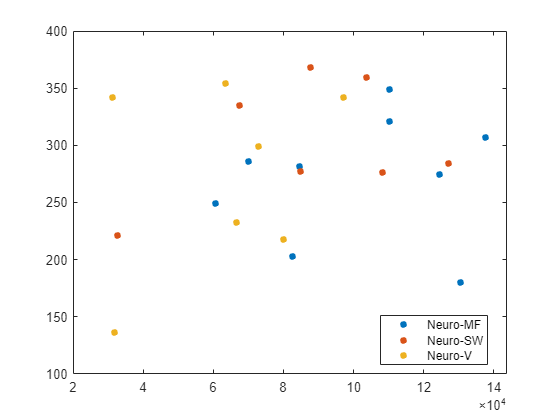


[rho,pval] = corr(data.TotalActivity, data.ClusterNAnteriorVentralAvg,'Type','Spearman')

rho = 0.1413

pval = 0.5185

## MF:

[rho,pval] = corr(dataMF.TotalActivity, dataMF.ClusterNAnteriorVentralAvg,'Type','Spearman')

rho = 0.1500

pval = 0.7081

## SW:

[rho,pval] = corr(dataSW.TotalActivity, dataSW.ClusterNAnteriorVentralAvg,'Type','Spearman')

rho = 0.2143

pval = 0.6615

## V:

[rho,pval] = corr(dataV.TotalActivity, dataV.ClusterNAnteriorVentralAvg,'Type','Spearman')

rho = -0.1429

pval = 0.7825

# ClusterN Anterior Dorsal Avg

gscatter(data.TotalActivity, data.ClusterNAnteriorDorsalAvg, data.Group)


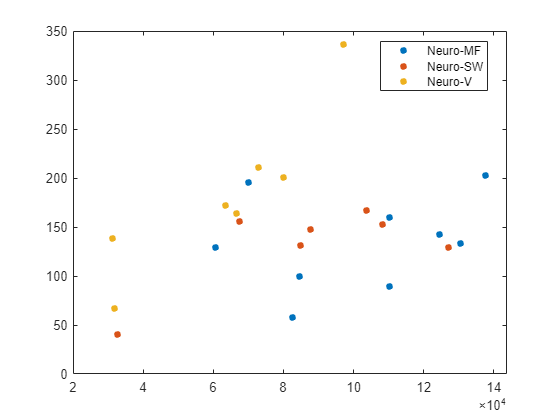


[rho,pval] = corr(data.TotalActivity, data.ClusterNAnteriorDorsalAvg,'Type','Spearman')

rho = 0.1517

pval = 0.4895

## MF:

[rho,pval] = corr(dataMF.TotalActivity, dataMF.ClusterNAnteriorDorsalAvg,'Type','Spearman')

rho = 0.4167

pval = 0.2696

## SW:

[rho,pval] = corr(dataSW.TotalActivity, dataSW.ClusterNAnteriorDorsalAvg,'Type','Spearman')

rho = 0.1786

pval = 0.7131

## V:

[rho,pval] = corr(dataV.TotalActivity, dataV.ClusterNAnteriorDorsalAvg,'Type','Spearman')

rho = 0.8929

pval = 0.0123

# ClusterN Posterior Avg

gscatter(data.TotalActivity, data.ClusterNPosteriorAvg, data.Group)


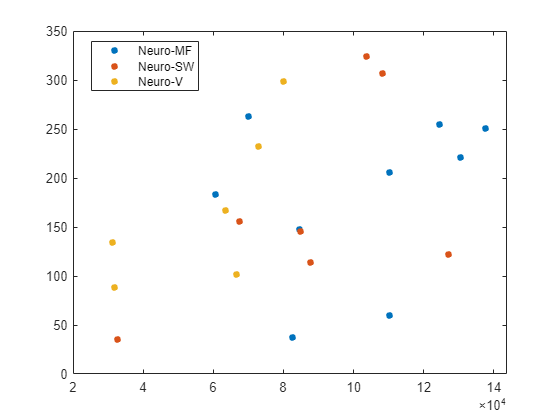


[rho,pval] = corr(data.TotalActivity, data.ClusterNPosteriorAvg,'Type','Spearman','Rows','complete')

rho = 0.3473

pval = 0.1137

## MF:

[rho,pval] = corr(dataMF.TotalActivity, dataMF.ClusterNPosteriorAvg,'Type','Spearman','Rows','complete')

rho = 0.3167

pval = 0.4101

## SW:

[rho,pval] = corr(dataSW.TotalActivity, dataSW.ClusterNPosteriorAvg,'Type','Spearman','Rows','complete')

rho = 0.3929

pval = 0.3956

## V:

[rho,pval] = corr(dataV.TotalActivity, dataV.ClusterNPosteriorAvg,'Type','Spearman','Rows','complete')

rho = 0.7143

pval = 0.1361
